# The characteristics of COVID-19 vaccine-related headache: Clues gathered from the healthcare personnel in the pandemic

**DOI:** 10.1177/03331024211042390

**Published:** 2021-09-12

**Authors:** Esme Ekizoglu, Haşim Gezegen, Pınar Yalınay Dikmen, Elif Kocasoy Orhan, Mustafa Ertaş, Betül Baykan

**Affiliations:** 1 Istanbul University, Istanbul Faculty of Medicine, Department of Neurology, Istanbul, Turkey; 2 Acıbadem Mehmet Ali Aydınlar University School of Medicine, Department of Neurology, Istanbul, Turkey

**Keywords:** COVID-19 vaccine, vaccination, vaccine, headache, adverse event

## Abstract

**Introduction:**

Headache is a frequent adverse event after viral vaccines. We aimed to investigate the frequency and clinical associations of COVID-19 vaccine-related headache.

**Methods:**

The characteristics, associations of this headache, main comorbidities, headache history following the influenza vaccine and during COVID-19 were investigated using a web-based questionnaire.

**Results:**

A total of 1819 healthcare personnel (mean age: 44.4 ± 13.4 years, 1222 females), vaccinated with inactivated virus, contributed to the survey; 209 (11.4%) had been infected with COVID-19. A total of 556 participants (30.6%) reported headache with significant female dominance (36.1% vs. 19.3%), 1.8 ± 3.5 (median: 1; IQR: 0–2) days following vaccination. One hundred and forty-four participants (25.9%) experienced headache lasting ≥3 days. Headache was mostly bilateral without accompanying phenomena, less severe, and shorter than COVID-19-related headache. The presence of primary headaches and migraine were significantly associated with COVID-19 vaccine-related headache (ORs = 2.16 [95% CI 1.74–2.68] and 1.65 [1.24–2.19], respectively). Headache during COVID-19 or following influenza vaccine also showed significant association with headache following COVID-19 vaccine (OR = 4.3 [95% CI 1.82–10.2] and OR = 4.84 [95% CI 2.84–8.23], respectively). Only thyroid diseases showed a significant association (OR = 1.54 [95% CI 1.15–2.08]) with vaccine-related headache among the common comorbidities.

**Conclusion:**

Headache is observed in 30.6% of the healthcare workers following COVID-19 vaccine and mostly experienced by females with pre-existing primary headaches, thyroid disorders, headache during COVID-19, or headache related to the influenza vaccine.

## Introduction

The coronavirus disease 2019 (COVID-19) was identified as a pandemic on 11 March 2020 (1), and has affected the whole world and caused the death of millions of people so far (2). Because of the lack of specific treatment and increased propagation rate due to new mutations, vaccination seems to be the best weapon to reduce morbidity and mortality in this pandemic (3). According to the World Health Organization (WHO), as of 14 May 2021, 184 candidate vaccines are in the preclinical evaluation and 100 vaccine candidates are under clinical evaluation to manage COVID-19, whereas 12 were authorized and approved for mass vaccination (4).

Headache is the most common neurological symptom, one of the most frequent problems in the COVID-19 pandemic and has a prevalence of 10–37% (5,6). Recent studies showed that COVID-19 related headache has some distinctive features (7). Furthermore, COVID-19 vaccine trials also disclosed that headache was among the most frequently reported systemic adverse events (8), being 39–80% with mRNA, 39–66% with adenovirus vectored and 13% with inactivated virus vaccines (9–13).

A recent study exploring real-life data on all adverse events related to mRNA COVID-19 vaccine reported headache following vaccination among 45.6% of 877 participants in the Czech Republic (14). Taking into account that the headache is also a frequent adverse event seen following other viral vaccines such as influenza (15), measles-mumps-rubella (16), varicella-zoster virus (VZV) (17) and human papillomavirus (HPV) (18), this latter finding is not surprising. However, the features and clinical correlates of COVID-19 vaccine-related headache are not well described yet. Therefore, we aimed to evaluate the characteristics and clinical associations of headaches experienced following COVID-19 vaccines and to understand their pathophysiological clues, based on a detailed survey in healthcare workers and medical students.

## Methods

After the COVID-19 vaccination, starting from healthcare professionals for the first time in January 2021 in our country, distinct headache properties caught our attention in consultation and the emergency department. We designed a cross-sectional, web-based questionnaire study to examine and understand this COVID-19 vaccine-related headache, concentrating on headache features, pre-existing headache, and previous vaccination history.

The questionnaire included a total of 61 questions and was based on seven different headlines (Supplement 1). The questionnaire headlines were a) vaccination type: Inactivated virus or mRNA vaccine (CoronaVac; Sinovac Life Sciences or Pfizer/Fosun Pharma/Biontech); b) socio-demographic variables (age, gender, occupation); c) medical history and the use of medications); d) COVID-19-related variables (severity of the disease, symptoms, COVID-related headache and its features); e) COVID-19 vaccine-related variables (other adverse effects, headache and its features, complaints accompanying headache); f) previous influenza vaccine-related headache; g) pre-existing primary headaches and related variables.

All related diagnostic points in the International Classification of Headache Disorders, third edition (ICHD-3) were asked in the questionnaire, as seen in the supplement, and a batch file was computed in the SPSS for the answers to the questions related to pre-existing headaches fulfilling the criteria according to ICHD-3 (19). Diagnoses of pre-existing definite migraine, probable migraine, definite tension-type headache (TTH) and probable TTH were made accordingly. The severity of headache after vaccination was questioned as being mild (not very disturbing and did not interfere with the daily work), moderate (uncomfortable but lets the participant do daily work) or severe (does not allow them to do daily work) (Supplement 1). This user-friendly questionnaire, accessible with a web-based link and also suitable for smartphones, was also tested for technical functionality. After the Ethics Committee (30.03.2021/156540) and the Ministry of Health department’s approval, vaccinated healthcare staff and students of the Istanbul Faculty of Medicine and Acibadem University Hospitals were invited to volunteer for the survey by text messages sent to their mobile phones in the name of the Head of Managements of both University Hospitals.

The healthcare workers were also invited via free social media platforms of the authors and their close circle (Whatsapp, Facebook, Twitter or Instagram). These invitations announced that a survey study investigating headache following COVID-19 immunization was planned and indicated the web-based link for volunteers. All invitees were informed about the aim of the survey as well as the length of time it would take, and eligible participants who accepted to fill out the survey – older than 18 years who had received at least one dose of a COVID-19 vaccine – were included in the study. A total of 2401 healthcare staff and 1632 students were invited and 1948 answered the survey. Surveys completed by 108 participants who were not vaccinated were excluded. Access to the web-based link, hence the data acquisition, was continued for 45 days. All questions were obligatory to answer and some questions were designed so the participant could choose only one situation. The survey could be submitted after filling out all questions to ensure completeness and could be filled out only once via the same mobile phone.

### Statistical analysis

Descriptive analyses were applied and normality was checked statistically. The demographic and clinical characteristics of the group with COVID-19 vaccine-related headache were compared to the groups a) without COVID-19 vaccine-related headache, b) with COVID-19 headache and c) with migraine or TTH. Chi-square and non-parametric Mann–Whitney U tests were used for the group comparisons; where appropriate, median values and interquartile ranges were reported accordingly. Univariate analyses were done using a binary logistic regression model, which was computed with the default “enter” method to reveal clinical characteristics suggesting headache related to the COVID-19 vaccine. Specific variables present in all participants were also compared by a multivariate analysis model with logistic regression (Supplement 2). IBM SPSS Statistics Version 22 was used and *p* < 0.05 was considered as statistically significant.

## Results

A total of 1840 participants contributed to our study and only 21 (1.1%) of them had received the mRNA based-vaccine. In this small group, nine participants (42.9%) reported vaccine-related headache. All of the remaining 1819 participants (1222 females, 67.2%) with a mean age of 44.4 ± 13.4 years were vaccinated with the inactivated virus; 1694 (93.1%) of them received both doses and 125 (6.9%) participants had had only the first dose. Among them, 1121 (61.6%) were physicians, 192 (10.6%) medical students, 180 (9.9%) nurses, and 326 (17.9%) other healthcare workers. Descriptive and statistical analyses were done in this latter group, who received the inactivated viral vaccine with alum as the adjuvant.

COVID-19 vaccine-related headache occurred in 556 (30.6%) participants, 1.8 ± 3.5 (median:1; IQR: 0–2) days later following vaccination. In this group, 244 participants (43.9%) experienced headache following the first dose, 139 (25%) following the second dose, whereas 173 (31.1%) experienced headache following both doses. Out of 125 participants who had only had the first dose of vaccine, 95 (5.2% of all participants) did not experience headache and the possibility of having headache following the second dose in this group remains unknown. Headache following COVID-19 vaccine lasted ≥3 days in 144 (25.9%) participants and <3 days in the remaining 412 (74.1%) participants; the headache had a duration <12 h in 259 (46.6%), between 12–24 hours in 86 (15.5%) and >24 h in 67 (12%) of those with vaccine-related headache. Among all participants, 441 out of 1222 females (36.1%) and 115 out of 597 males (19.3%) had experienced headache following vaccination, which showed a significant female dominance in the group having COVID-19 vaccine-related headache (*p* < 0.001, OR = 2.37 [95% CI 1.87–2.99] in univariate analyses) (Table 1). Clinical and demographical characteristics of participants with and without COVID-19 vaccine-related headache are shown in Table 1, comparatively. A total of 897 healthcare workers (48.8%) were using medications for comorbid diseases.

**Table 1.** Clinical characteristics of the participants with and without COVID-19 vaccine-related headache.

	Study group with Sinovac vaccination					
	With COVID-19 vaccine-related headachen = 556 (30.6%)	Without COVID-19 vaccine-related headachen = 1263 (69.4%)	Totaln = 1819	*p*	OR	95% CI
Age, year, mean (SD)	43.4 (12.3)	44.9 (13.9)	44.4 (13.4)	**0.034** ^a^		
Sex						
Female n (%)	441 (79.3)	781 (61.8)	1222 (67.2)	**<0.001** ^c^	1.96	1.54–2.51
Male n (%)	115 (20.7)	482 (38.2)	597 (32.8)			
Comorbidities						
Hypertension n (%)	90 (16.2)	175 (13.8)	265 (14.6)	0.19	1.2	0.91–1.58
Diabetes n (%)	45 (8.1)	78 (6.2)	123 (6.8)	0.13	1.34	0.91–1.96
Thyroid diseases n (%)	97 (17.5)	130 (10.3)	157 (8.6)	**<0.004** ^c^	1.54	1.15–2.08
Hyperlipidemia n (%)	19 (3.4)	41 (3.2)	60 (3.3)	0.85	1.05	0.61–1.83
Cardiac diseases n (%)	9 (1.6)	33 (2.6)	42 (2.3)	0.2	0.61	0.29–1.29
Asthma n (%)	20 (3.6)	39 (3.1)	59 (3.2)	0.57	1.17	0.67–2.03
Pre-existing primary headaches n (%)	377 (67.8)	594 (47.0)	971 (53.4)	**<0.001** ^c^	2.16	1.74–2.68
Definite migraine, n (%)	131 (23.6)	145 (11.5)	276 (15.2)	**<0.001** ^b^	1.65	1.24–2.19
Definite tension-type headache, n (%)	134 (24.1)	204 (16.1)	338 (18.6)	**<0.001** ^b^	1.05	0.8–1.38
Allodynia in previous headaches, n (%)^†^	149 (39.5)	211 (35.5)	360 (37.1)	0.21	1.19	0.9–1.55
Headache days/month, median (IQR)	4 (3–5)	4 (3–5)	4 (3–5)	0.21		
Adverse events related to COVID-19 vaccine^††^						
Fever n (%)	54 (9.7)	32 (2.5)	86 (4.7)	**<0.001** ^c^	3.99	2.44–6.5
Fatigue n (%)	279 (20.2)	216 (17.1)	495 (27.2)	**<0.001** ^b^	4.88	3.9–6.1
Myalgia n (%)	190 (34.2)	200 (15.8)	390 (21.4)	**<0.001** ^b^	2.76	2.19–3.48
Joint pain n (%)	103 (18.5)	66 (5.2)	169 (9.3)	**<0.001** ^b^	4.12	2.97–5.72
Itching n (%)	20 (3.6)	20 (1.6)	40 (2.2)	**0.009** ^b^	2.32	1.24–4.35
Dyspnea n (%)	4 (0.7)	7 (0.5)	11 (0.6)	0.68	1.3	0.38–4.46
Nausea/vomiting n (%)	38 (6.8)	30 (2.4)	68 (3.7)	**<0.001** ^b^	3.01	1.85–4.92

Note: Numbers in bold are statistically significant values.^a^Independent Samples Test.

^b^Univariate logistic regression.

^c^Multivariate logistic regression model including female gender, the presence of primary headache, thyroid disorder and the occurrence of fever as COVID-19 vaccine adverse event.

^†^Among 971patients with primary headaches.

^††^1081participants (59.4%) did not report any adverse event.

IQR: interquartile range (Q1–Q3).

Among the responders to the survey, 207 had also been diagnosed with COVID-19, and 134 (64.7%) of these had experienced headaches during infection. Among this group, only one participant had COVID-19 after receiving the vaccine during the study period, she reported having COVID-19 related headache but not vaccine-related headache. COVID-19 related headache was more frequently seen in the group with COVID-19 vaccine-related headache (42 out of 49 participants, 85.7%) in comparison to the group without COVID-19 vaccine-related headache (92 out of 158 participants, 58.2%) (*p* < 0.001, OR = 4.3 [95% CI 1.82–10.2]). Furthermore, severe COVID-19 was reported more frequently in the group experiencing COVID-19 vaccine-related headache (seven out of 49 participants, 14.3%) in comparison to those without COVID-19 vaccine-related headache (17 out of 158 participants, 10.8%) without reaching statistical significance.

**Figure 1.**
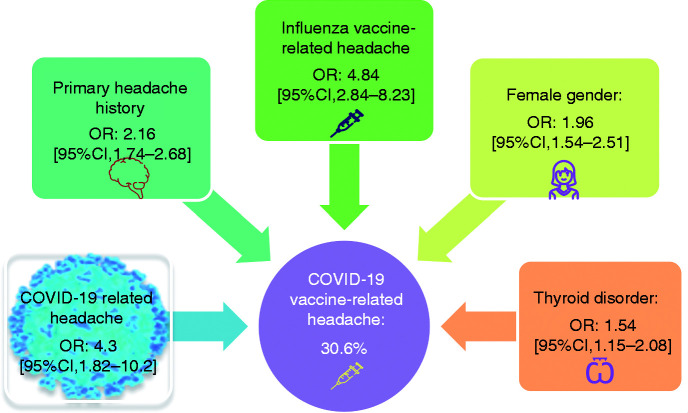
Odds ratios of clinical features associated with COVID-19 vaccine-related headache.

Additionally, 864 participants (47.5%) had been vaccinated for influenza in the past; among these, 65 (7.5%) had experienced headache after this vaccine, and 799 did not have any headache following the influenza vaccine, whereas 266 did not remember if they experienced headache or not. Headache following flu vaccine was more significantly reported in those with COVID-19 vaccine-related headache (42 out of 261 participants, 16.1%) compared to those without COVID-19 vaccine-related headache (23 out of 603 participants, 3.8%) (*p* < 0.001, OR = 4.84 [95% CI 2.84–8.23]) (Figure 1).

The quality of headache related to COVID-19 vaccine disclosed heterogeneous features: It was described as throbbing by 223 (40.1%), pressing by 169 (30.4%), jolting by 328 (59%), fiery by 16 (2.9%), stabbing by 53 (9.5%) and/or pricking by 29 (5.2%) participants, whereas 22 (3.9%) subjects reported not having any specific headache quality. Headache characteristics and response to analgesics of the vaccine-related headache are given in Table 2. Headache improved without analgesics in 171 (30.8%) subjects. Drugs used for pain relief by the remaining 385 (69.2%) participants were as follows: paracetamol (n = 281, 73%), nonsteroidal anti*-*inflammatory drugs (n = 219, 56.9%), ergots (n = 4, 1%), triptans (n = 7, 1.8%), myorelaxants (n = 20, 5.2%) and metamizol (n = 5, 1.3%). There were only seven subjects who had to be admitted to the emergency department due to this headache following vaccination, five were diagnosed with another medical condition. Clinical features, according to the severity of COVID-19 vaccine-related headache, are given in Table 3. Other COVID-19 vaccine-related adverse events in addition to headache were also investigated and are given in Table 1.

**Table 2.** Comparative characteristics of COVID-19 vaccine-related and COVID-19 related headaches in the healthcare workers.

	COVID-19 vaccine-related headachen = 556 (30.6%)^†^	COVID-19 related headachen = 134 (64.7%)^††^	p*	OR	95% CI
Headache location					
Unilateral/more prominent on one side n (%)	184 (33.1)	27 (20.2)	**0.004**	1.96	1.24–3.1
Bilateral n (%)	372 (66.9)	107 (79.8)			
Accompanying symptoms					
Anosmia n (%)	4 (0.7)	73 (52.5)	**<0.001**	165.15	58.33–467.53
Ageusia n (%)	5 (0.9)	65 (48.5)	**<0.001**	103.81	40.41–266.66
Aggravation with physical activity n (%)	137 (24.6)	61 (45.5)	**<0.001**	2.56	1.73–3.78
Phonophobia n (%)	83 (14.9)	24 (17.91)	0.39	1.24	0.75–2.05
Photophobia n (%)	94 (16.9)	39 (29.1)	**0.002**	2.02	1.31–3.11
Nausea n (%)	67 (12.1)	27 (20.15)	**0.015**	1.84	1.15–3.02
Osmophobia n (%)	22 (4)	7 (5.22)	0.51	1.34	0.56–3.2
None n (%)	198 (35.61)				
Severity of headache			**<0.001**		
Mild n (%)	126 (22.7)	23 (17.1)			
Moderate n (%)	370 (66.5)	79 (59)			
Severe n (%)	60 (10.79)	32 (23.9)			
Duration of headache, days					
Median (IQR)	0.5 (0.2–3)	4 (2.7–7)	**<0.001**		
Use of analgesics n (%)**			**0.001**		
No response to analgesics n (%)	12 (2.2)	15 (11.2)			
Partial response to analgesics n (%*)*	241 (43.3)	78 (58.1)			
Completely recovered with analgesics % n (%)	132 (23.7)	41 (30.6)			

Note: Numbers in bold are statistically significant values.*Univariate logistic regression, Mann–Whitney U or Chi-square.

**385 participants (69.2%) with COVID-19 vaccine-related headache and all of those with COVID-19 related headache had used analgesics.

^†^Among all of 1819 participants.

^††^Among 207 participants, who had been diagnosed with COVID-19.

IQR: interquartile range (Q1–Q3).

**Table 3.** Clinical features according to severity of COVID-19 vaccine-related headache.

	Mild vaccine related headachen = 126	Moderate vaccine related headachen = 370	Severe vaccine related headachen = 60	p* (1 vs. 3)	p* (2 vs. 3)
Vaccine-related headache during ≥3 days, n (%)	19 (15.1)	105 (28.4)	20 (33.3)	**0.007**	0.43
Vaccine-related headache during <3 days, n (%)	107 (84.9)	265 (71.6)	40 (66.6)		
Associated fever, n (%)	13 (10.3)	38 (10.3)	3 (5)	0.59	0.24
Pre-existing primary headaches, n (%)	70 (55.5)	261 (70.5)	46 (76.7)	**0.005**	0.33
Definite migraine, n (%)	16 (12.7)	92 (24.9)	23 (38.3)	**<0.001**	**0.029**
Duration between COVID-19 and vaccination, months, median (IQR)**	5.5 (3–9)	3 (2.25–6.5)	4 (2–5)	0.41	0.89

Note: Numbers in bold are statistically significant values.*Pearson Chi-Square, Fisher’s Exact test or Mann–Whitney U.

IQR: interquartile range (Q1–Q3).

**18, 28 and three participants reported mild, moderate and severe vaccine related headache, respectively.

A multivariate logistic regression model showed that the presence of primary headache, thyroid disorder, the occurrence of fever as a COVID-19 vaccine adverse event, and having female gender were significant variables suggesting the emergence of headache related to COVID-19 vaccine (*p* = 0.004 for thyroid disorder and *p* < 0.001 for others) (Table 1 and Supplement 2).

**Figure 2.**
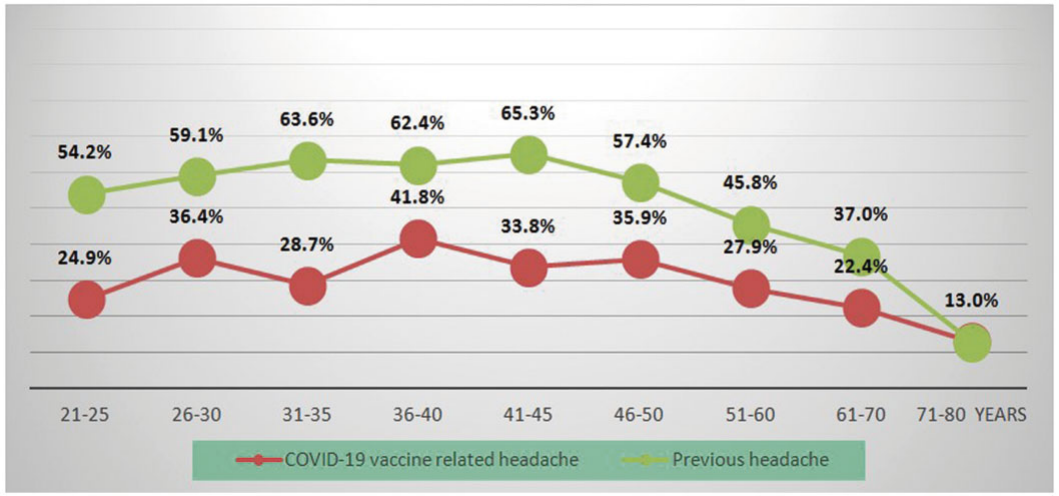
Percentages of the patients with vaccine-related headache and previous headache within the age groups.

According to the responses of the participants to the survey, 276 participants fulfilled the criteria of definite migraine, 170 probable migraine, 338 definite TTH, and 19 probable TTH. The rates of the presence of COVID-19 vaccine-related headache and previous headaches were calculated within each age block of 5–10 years separately and showed a decrease with older age in participants with COVID-19 vaccine-related headache similarly to those with primary headaches (Figure 2). Comparative characteristics of COVID-19 vaccine-related headache with migraine and TTH are given in Table 4.

**Table 4.** Comparative characteristics of COVID-19 vaccine-related headache and primary headache disorders in the healthcare workers.

	(1)COVID-19 vaccine-related headachen = 556	(2)Migraine(definite or probable)n = 446	(3)TTH(definite orprobable)n=357	*p* (1 vs. 2)*	*p* (1 vs. 3)*
Headache location					
Unilateral/more prominent on one side n (%)	184 (33.1)	305 (68.4)	141 (39.5)	**<0.001**	**0.06**
Bilateral n (%)	372 (66.9)	141 (31.6)	216 (60.5)		
Accompanying symptoms					
Aggravation with physical activity n (%)	137 (24.6)	366 (82.1)	170 (47.6)	**<0.001**	**<0.001**
Phonophobia n (%)	83 (14.9)	307 (68.3)	72 (20.2)	**<0.001**	0.05
Photophobia n (%)	94 (16.9)	335 (75.21)	43 (12)	**<0.001**	0.05
Nausea n (%)	67 (12.1)	266 (59.6)	11 (3.1)	**<0.001**	**<0.001**
Osmophobia n (%)	22 (4)	119 (26.7)	8 (2.2)	**<0.001**	0.2
None n (%)	198 (35.61)	20 (4.5)	242 (67.8)	**<0.001**	**<0.001**
Severity of headache					
Mild n (%)	126 (22.7)	26 (5.8)	77 (21.6)	**<0.001**	**<0.001**
Moderate n (%)	370 (66.5)	326 (73.1)	271 (75.9)		
Severe n (%)	60 (10.79)	94 (21.1)	9 (2.5)		
Duration of headache, days					
Median (IQR)	0.5 (0.2–3)	0.3 (0.2–0.7)	0.2 (0.1–0.3)	**<0.001**	**<0.001**

Note: Numbers in bold are statistically significant values.TTH: Tension type headache.

*Mann-Whitney U or Pearson Chi-square.

IQR, Interquartile range (Q1–Q3)

## Discussion

In this study, nearly one-third of participants (30.6%) reported headache after COVID-19 inactivated virus vaccine. This headache condition showed different features in comparison to COVID-19 related headache and previous headache disorder. Typical symptoms such as anosmia/ageusia for COVID-19 related headache as well as osmophobia, phono- and photophobia for migraine were less frequently accompanying COVID-19 vaccine-related headache. This particular headache showed heterogeneous character, was mostly bilateral, less severe and shorter (lasted 0.5; 0.2–3 days, median; IQR) than COVID-19 related headache experienced by the same population (Table 2). Furthermore, diagnosis of migraine or tension-type headache, history of influenza vaccine-related headache or COVID-19 related headache, and thyroid disorders were more frequently seen in the group experiencing headache following vaccination.

Headache related to vaccination has been reported with various types of vaccines at different rates: After adenovirus-vectored Ebola virus vaccine in 46% (20), inactivated influenza vaccine in 31.8% (21) and oral influenza H1N1 vaccine in 7% (22). Headache is also one of the most commonly reported symptoms in 33.9% of the 9-valent human papillomavirus vaccine (9vHPV) as an adverse event after its approval (23). The eradication of smallpox represents an important example of the lasting benefit of vaccinations for human health and headache has been reported in 44% of recipients of the smallpox vaccine (24). The headache following smallpox vaccination was generally transient but might also have been severe, needing hospitalization (25).

In the COVID-19 era, diverse types of vaccines against this virus, with different advantages and disadvantages, have been recently developed including inactivated virus, nucleic acid, adenovirus-based vector, and recombinant subunits vaccines (26). The frequency of headache after vaccination showed a wide range in clinical trials with different vaccines: 39–80% with mRNA, 39–66% with adenovirus vectored and 13% with inactivated virus vaccines (9–13). Furthermore, pain at the site of injection, fever, myalgia, and fatigue are reported as other common adverse events following COVID-19 vaccines (8). We found a significant co-occurrence between the headache and other adverse events, such as fever, fatigue, myalgia, joint pain, itching, and nausea/vomiting, suggesting a common predisposition in the group with the vaccine-related headache.

It is interesting to note that COVID-19 vaccine-related headache itself has distinctive characteristics when compared to headache related to COVID-19 in the same group of healthcare workers, as seen in Table 2. Besides being less severe, with an improvement without analgesics in almost one-third of subjects, and shorter than with the real infection with SARS-CoV-2, vaccine-related headache with inactivated virus appeared more significantly in females, according to our results. Even though this is not unexpected given the female preponderance in migraine, the headache related to COVID-19 itself had shown a contrasting male predilection, previously (7,27). Previous studies investigating the safety of inactivated influenza vaccine also reported that vaccine-related headache was more common in women and younger people, similar to our findings (21). Moreover, patients with headache related to COVID-19 and mRNA COVID-19 vaccine were also found to be younger in comparison to those without headache (9,28). The comparison of characteristics of vaccine-related headaches with pre-existing migraine and TTH separately revealed that this particular headache lasts longer than these primary headaches, as seen in Table 4. The characteristics of vaccine-related headache most resembled TTH, being bilateral and mostly without any associated features, and its severity was placed in between migraine and TTH.

The COVID-19-related headache showed a very high incidence of anosmia/ageusia in line with previous research (28–30) whereas, intriguingly, these symptoms were almost absent in vaccine-related headache triggered with the inactivated virus vaccine. The underlying mechanisms of headache related to COVID-19 are not yet uncovered (31). This unique relation between the headache and anosmia/ageusia, which does not appear in vaccine-related headache, may give us some clues and support a direct invasion of the cranial nerves by the virus during the airway entry.

The underlying mechanisms of the vaccine-related headache are also not illuminated so far. Regarding its characteristics, the bilateral jolting, pressing, or throbbing headache of moderate/severe intensity, commonly in association with fever that has occurred in clear temporal relation with vaccination, comes to the front. Moreover, vaccine-related headaches showed very low rates of all accompanying symptoms such as osmophobia, phono- and photophobia, showing that prominent sensory system activation is not involved in this plain headache process. Supporting that, previous allodynia indicating central sensitization is not an effective marker in the development of vaccine-related headache according to our results.

Vaccination related-headache showed an overall significant association with fever, as well as, with other pain conditions, including joint pain and myalgia (Table 1), which may indicate a shared immunogenic or genetic propensity. Fever was also found to be a headache associated symptom in COVID-19. A recent study investigating headache characteristics in relation to COVID-19 reported that fever was more frequently seen in patients with headache than those without headache among 60 age/gender-matched COVID-19 patients (28).

Vaccination induces protective immunity through different immunological mechanisms. Viral proteins of COVID-19 and their interactions with host factors elicit the production of pro-inflammatory cytokine levels (26,32). Vaccines usually contain adjuvants, which themselves are also sensed by the immune system and have the potential to promote the immune response (33). The COVID-19 vaccine mostly used in our country during this study was an inactivated viral vaccine including alum, an aluminum salt–based adjuvant. The mechanisms by which alum induces immune responses are not completely understood. Several studies have demonstrated that alum signals through the NLRP3 inflammasome, leading to caspase 1-dependent release of the pro-inflammatory cytokines IL-1β and IL-18 (33). Furthermore, a previous study of the smallpox vaccine showed a higher frequency of headache among primary vaccine recipients than revaccinated subjects and increased serum levels of cytokines in the group receiving the first dose (34). In our study, headache was also more frequently reported following the first dose (43.9%) than the second dose (25%), a finding that suggests a stronger immune response against this novel antigen. Therefore, a pro-inflammatory state might be a predominant situation following vaccination, similar to that in COVID-19 related headache, which was reported to associate with high levels of pro-inflammatory cytokines (35). The history of COVID-19 related headache, migraine, and the presence of fever seen more frequently in subjects with COVID-19 vaccine-related headache, may suggest the role of inflammation lying behind the development of headache related to vaccination. Moreover, the lack of an association between vaccine-related headache and allodynia may indicate that headache is not promoted by hyperexcitability in this condition.

We also found a significant relationship between headache development as a side effect after COVID-19 vaccination and history of previous thyroid disease in healthcare workers, although the difference was modest. A high prevalence of thyroid dysfunction in general and specifically hypothyroidism was reported among patients with primary headaches. Albeit there are some contradictory results, a possible bidirectional relationship between migraine and hypothyroidism was suggested, which may indicate a common either genetic or autoimmune mechanism underlying both diseases (36). Moreover, a recent study detected hormonal changes compatible with the hypoactive hypothalamus-pituitary-thyroid axis in 51 patients with chronic tension-type headache (37). The hypothalamus controls the brain-hormonal interface, including the thyroidal and sex hormones. Furthermore, this structure regulates the body temperature besides its various neuroanatomical connections to pain-modulating systems (38). In this study, in addition to the associations between vaccine-related headache and female gender, primary headache disorders and fever, a higher frequency of thyroid dysfunction along with the lack of similar associations of previous common disorders (like those indicating vascular risk factors) could be interpreted as a clue to that the hypothalamus may serve for the vaccine-related headache generation. This COVID-19 vaccine-related headache seems to occur in subjects with a genetic propensity or immunogenicity via a particular pain-generating mechanism, probably involving the hypothalamus. However, our results, based mainly on self-reports, should be interpreted with caution even though participants were healthcare staff. This preliminary result should be investigated with future prospective studies after vaccination along with laboratory measurements.

Our study emphasized the lack of an appropriate place for the vaccine-related headache in the International Classification of Headache Disorders-3 classification (19). The most convenient title is *8.1 Headache attributed to the use of or exposure to a substance*. Given the increasing importance of mass vaccinations worldwide, there is an urgent need for a special place, which is already deserved, for the vaccine-related headache in our headache classification.

## Limitations and strengths of the study

One of the limitations of this study was that the headache characteristics were investigated via questionnaire with the potential of a reporting bias well known in all survey studies. Elder retired healthcare workers and those younger than 18 years were not included in this study, which also caused a selection bias limiting the generalizability of the study for the general population. Furthermore, we aimed to investigate in detail all possible qualities of headaches but some headache descriptions such as jolting, fiery, and pricking were not totally in line with those employed in ICHD criteria, leading to some difficulty in terms of classification. Additionally, we could not investigate laboratory markers with a potential relationship with headache conditions and the diagnosis was not clear in a few patients admitted to the hospital due to headache.

Our study targeted a restricted population and most of them were physicians. But this point may also constitute a strength of our study. The fact that all participants were healthcare workers may increase the reliability of the study. Moreover, we used a detailed dedicated questionnaire investigating various characteristics of previous headaches and also COVID-19 related headaches in the same group.

## Conclusion

We investigated the headache related to COVID-19 inactivated virus vaccine and found that this frequent adverse event with a prevalence of 30.6% was mostly experienced by females, and was significantly associated with pre-existing primary headaches, thyroid disorders, headaches during COVID-19 course, or headache related to the influenza vaccine. Therefore, we suggest that it may be reasonable to give appropriate counselling before vaccination to these people and to use prophylactic simple analgesic drugs if needed. Headache was mostly bilateral without accompanying phenomena, less severe, and shorter than COVID-19 related headache, and associated with fever and other vaccine-related adverse events. Our findings also suggested that COVID-19 vaccine-related headache was a different and simple secondary headache entity, in comparison to COVID-19 related headache and also individuals’ pre-existing primary headaches.

## Key findings

- Nearly one-third of all participants (556; 30.6%) reported COVID-19 vaccine-related headache following vaccination with inactivated virus.
- COVID-19 vaccine-related headache was mostly experienced by females, and significantly associated with pre-existing primary headaches, thyroid disorders, headache during COVID-19, or headache related to the influenza vaccine.
- Emerging headache after the COVID-19 vaccine was mostly bilateral, without any accompanying phenomena, less severe and shorter than the COVID-19 related headache (0.5; 0.2–3 vs. 4; 2.7–7 days, median; IQR); associated with fever and other adverse events.

## Supplemental Material

sj-pdf-1-cep-10.1177_03331024211042390 - Supplemental material for The characteristics of COVID-19 vaccine-related headache: Clues gathered from the healthcare personnel in the pandemic Click here for additional data file. Supplemental material, sj-pdf-1-cep-10.1177_03331024211042390 for The characteristics of COVID-19 vaccine-related headache: Clues gathered from the healthcare personnel in the pandemic by Esme Ekizoglu, Haşim Gezegen, Pınar Yalınay Dikmen, Elif Kocasoy Orhan, Mustafa Ertaş and Betül Baykan in Cephalalgia

sj-pdf-2-cep-10.1177_03331024211042390 - Supplemental material for The characteristics of COVID-19 vaccine-related headache: Clues gathered from the healthcare personnel in the pandemic Click here for additional data file. Supplemental material, sj-pdf-2-cep-10.1177_03331024211042390 for The characteristics of COVID-19 vaccine-related headache: Clues gathered from the healthcare personnel in the pandemic by Esme Ekizoglu, Haşim Gezegen, Pınar Yalınay Dikmen, Elif Kocasoy Orhan, Mustafa Ertaş and Betül Baykan in Cephalalgia
