# Supplementary material for: The characteristics of COVID-19 vaccine-related headache: Clues gathered from the healthcare personnel in the pandemic
Source: Cephalalgia. 2021 Sep 12;42(4-5):366–75. doi: 10.1177/03331024211042390 (PMC8988457; doi:10.1177/03331024211042390)
Supplement: sj-pdf-1-cep-10.1177_03331024211042390 - Supplemental material for The characteristics of COVID-19 vaccine-related headache: Clues gathered from the healthcare personnel in the pandemic [file sj-pdf-1-cep-10.1177_03331024211042390.pdf]

Supplement 2: Multivariate analyses including risk factors available in all group of 1819 participants

Variables	B	S.E.	Wald	df	Sig.	Exp(B)	95% C.I.for EXP(B)	
							Lower	Upper
Pre-existing primary headache	.772	.110	49.178	1	.000	2.164	1.744	2.685
Thyroid disorder	.435	.152	8.145	1	.004	1.544	1.146	2.082
Fever as COVID-19 vaccine adverse event	1.384	.250	30.736	1	.000	3.990	2.446	6.507
Female gender	.676	.125	29.127	1	.000	1.965	1.538	2.512
Constant	-1.872	.122	236.152	1	.000	.154		

B: beta (regression coefficient); SE, standard error; d.f., degree for freedom; Sig., significance; Exp (B), OR: exponential B, odds ratio. (This model classified 70.8% of all cases correctly with a cut value of .05)
