# Supplementary material for: The characteristics of COVID-19 vaccine-related headache: Clues gathered from the healthcare personnel in the pandemic
Source: Cephalalgia. 2021 Sep 12;42(4-5):366–75. doi: 10.1177/03331024211042390 (PMC8988457; doi:10.1177/03331024211042390)
Supplement: sj-pdf-2-cep-10.1177_03331024211042390 - Supplemental material for The characteristics of COVID-19 vaccine-related headache: Clues gathered from the healthcare personnel in the pandemic [file sj-pdf-2-cep-10.1177_03331024211042390.pdf]

Covid-19 Vaccine and Headache Questionnaire

Dear Participant;
We are doing a research on the relationship between headache and vaccine against to the new type of Covid-19 known as Coronavirus, which affects our country as well as the whole world. The information we will obtain will shed light on our better understanding of this important issue and the right intervention at the right point. We thank you very much for your contribution to science by participating in this questionnaire consisting of 61 questions and completed in approximately 8 minutes.

* Obligatory fields

1. Have you been vaccinated for Covid-19? *

- Yes
- No

Covid-19 Vaccine

2. Which vaccine did you get? *

- Pfizer/Biontech
- AstraZeneca
- Sinovac
- Moderna

3. How many times have you been vaccinated? *

- First dose
- Both doses

4. How old are you? *

____

5. What is your gender? *

- Female
- Male

6. What is your profession? *

- Physician
- Dentist
- Medical faculty student
- Dentistry student
- Nurse
- Laborant
- Pharmacist
- Technician
- Other health staff

7. Do you have any chronic illness? *

- [ ] Yes
- [ ] No

Chronic Illnesses

8. What is your chronic illness (you can choose more than one) ? *

- [ ] Hypertension
- [ ] Diabetes
- [ ] Ischemic heart disease
- [ ] Hyperlipidemia
- [ ] COPD
- [ ] Thyroid disease
- [ ] Depression
- [ ] Anxiety disorder
- [ ] Liver failure
- [ ] Digestive system disease
- [ ] Cancer
- [ ] Muscle disease
- [ ] Previous paralysis (with or without sequelae)
- [ ] Allergy
- [ ] Asthma
- [ ] None

Medications

9. Do you take any medication on a daily basis, regularly (you can choose more than one)? *

- ☐ Analgesics
- ☐ Anti-diabetic medication
- ☐ Thyroid medication
- ☐ Digestive system medication
- ☐ Anti-rheumatism medication
- ☐ Vitamin
- ☐ Heart or blood pressure medication
- ☐ Antiaggregants
- ☐ Lung medication
- ☐ Migraine medication
- ☐ Antidepressive drugs
- ☐ Sleeping pill
- ☐ None

10. Have you been diagnosed with Covid-19? *

- ◯ Yes
- ◯ No

Covid-19 Course

11. How was your clinical course of Covid-19? *

- ◯ Asymptomatic
- ◯ Home treatment with mild symptoms
- ◯ Hospitalization
- ◯ Intensive care admission

12. Which complaints did you have during the Covid-19 (you can choose more than one)? *

- [ ] Sore throat
- [ ] Cough
- [ ] Shortness of breath
- [ ] Inability to smell
- [ ] Inability to taste
- [ ] Lung involvement
- [ ] Nausea
- [ ] Vomiting
- [ ] Diarrhea
- [ ] Weakness
- [ ] Joint pain
- [ ] Muscle pain
- [ ] Chest pain
- [ ] None

13. Have you had an antibody test after Covid-19 ? *

- ( ) I have not
- ( ) Yes I have, positive
- ( ) Yes I have, negative

14. Did you suffer from a headache during Covid-19? *

- ( ) Yes
- ( ) No

Headache During Covid-19 Infection

15. How was your headache during Covid-19 ? (you can choose more than one) *

- [ ] One side of the head
- [ ] Both sides of the head

16. What was the character of your headache during Covid-19 (you can choose more than one)? *

- [ ] Throbbing
- [ ] Compressing
- [ ] Jolting
- [ ] Others

17. Did movements increase your headache during Covid-19 (e.g. leaning forward, climbing stairs) *

- ( ) Yes
- ( ) No

18. How was the severity of your headache during Covid-19? *

- ( ) Mild, non-irritating and did not interfere with my daily work
- ( ) Moderate, uncomfortable but I was able to do my daily work
- ( ) Severe I couldn't do my daily work

19. Which of them were accompanying your headache during Covid-19 (you can choose more than one)? *

- [ ] Nausea
- [ ] Vomiting
- [ ] Light sensitivity
- [ ] Sound sensitivity
- [ ] Discomfort from smell
- [ ] None

20. Did your headache during Covid-19 infection resolve after analgesics? *

- ( ) The pain was completely resolved
- ( ) The pain was relieved
- ( ) Analgesics did not help

21. How many DAYS of your headache lasted during or after Covid-19 infection? *

____

After Covid-19 Infection

22. How many months after Covid-19 infection you were vaccinated? *

____

Post-vaccination Headache

23. Have you experienced any side effects after Covid-19 vaccination (you can choose more than one)? *

- ☐ Fatigue
- ☐ Muscle pain
- ☐ Joint pain
- ☐ Fever (over 38 degrees)
- ☐ Mild fever (38 degrees or below)
- ☐ Itching
- ☐ Shortness of breath
- ☐ Nausea / vomiting
- ☐ None

24. Did you suffer from a headache after Covid-19 vaccine? *

- ◯ Yes
- ◯ No

Post-vaccination Headache

25. After which dose did you have a headache? (Answer according to the longest headache) *

- ◯ 1. dose
- ◯ 2. dose
- ◯ After both doses

26. How many days after the Covid-19 vaccination your headache started (if it started on the same day with the vaccine, enter 0 as a number)? *

27. How long did your headache last? *

- Two days or less
- Three days or longer

Post-vaccination Headache Duration (HOURS)

28. How many HOURS did the headache after vaccination last? *

Post-vaccination Headache Duration (DAYS)

29. How many DAYS did your headache after vaccination last? *

Post-vaccination Headache

30. How was the character of this headache (you can choose more than one)? *

- [ ] Throbbing
- [ ] Pressing
- [ ] Jolting
- [ ] Flammable
- [ ] Stabbing
- [ ] None

31. Where was your headache located (you can choose more than one)? *

- [ ] One side
- [ ] Both sides, more dominant on one side
- [ ] Both sides, same severity on both sides

32. Where was the headache most intense? *

- ( ) Behind or around the eye
- ( ) Back of the neck
- ( ) Face
- ( ) Vertex of the head
- ( ) Temporal regions

33. How was the severity of headache after vaccination? *

- ( ) Mild, non-irritating and did not interfere with my daily work
- ( ) Moderate, uncomfortable but I was able to do my daily work
- ( ) Severe, I couldn't do my daily work

34. How many times a day did you have to take medication for your headache (If you did not take any medication, enter zero)? *

35. How did your post-vaccination headache improved? *

- Got better without medication
- Completely improved with medication
- Decreased with medication
- Continued despite medication (I didn't go to the emergency room)
- I went to the emergency room/hospital because of headache

In Hospital

36. Which is the etiology for your headache, determined in the emergency room or hospital? *

- Vaccine-related headache
- Meningitis
- Encephalitis
- General infection
- Others (open ended)

Post-vaccination Headache

37. Which analgesics did you use to prevent or relieve your pain (you can choose more than one) ? *

- ☐ Paracetamol containing medicines (Parol, Minoset, Geralgine...)
- ☐ Anti-rheumatic painkillers (Apranax, Majezik, Arveles, Brufen, Advil, Etol...)
- ☐ Aspirin
- ☐ Ergotamine (Avmigran, Cafergot, Ergafein)
- ☐ Triptan (Migrex, Relpax, Imigran)
- ☐ Muscle relaxants (Muscoril, Sirdalud and similar)
- ☐ None

38. How much has your pain changed with this medication? *

- ◯ Not relieved
- ◯ Less than half relieve for the pain
- ◯ More than half relieve for the pain
- ◯ Completely relieved

39. Was this headache accompanied by one or more of the following (you can choose more than one)? *

- ☐ Fever / malaise
- ☐ Flu symptoms, runny nose or sore throat
- ☐ Stinging, burning or tearing in the eyes
- ☐ Nausea or vomiting
- ☐ Smells bothered me
- ☐ Light bothered me
- ☐ Sound bothered me
- ☐ Inability to smell
- ☐ Inability to taste
- ☐ Worsening with physical activityd (bending, climbing stairs)
- ☐ None

Pre-vaccination Headache

40. Have you ever had a headache after influenza (flu) vaccine? *

- Yes
- No
- I do not remember
- I've never had an influenza (flu) vaccine

41. Did you have a headache in 1 year before Covid-19 vaccine? *

- Yes
- No

Headache Within 1 Year Before Covid-19 Vaccination

42. How many DAYS of headache did you have in 1 year before Covid-19 vaccination? *

- Less than a day per month
- One day or more per month

Headache Within 1 Year Before Covid-19 Vaccination

43. How many DAYS PER MONTH have you had headaches in the previous year before Covid-19 vaccination? *

____

Headache Within 1 Year Before Covid-19 Vaccination

44. How long have you had this type of headache before Covid-19 vaccination? *

- One year or less
- More than one year

Headache Within 1 Year Before Covid-19 Vaccination

45. How many MONTHS you have this type of headache before vaccination? *

____

Headache Within 1 Year Before Covid-19 Vaccination

46. How many YEARS did you have this type of headache before the vaccination? *

____

Headache Within 1 Year Before Covid-19 Vaccination

47. How long your headache would usually last if you did NOT take attack medication for headache? *

- Seconds
- 4 seconds-less than 2 minutes
- 2 minutes-less than 30 minutes
- 30 minutes or 3 days maximum
- 4 days or longer

Headache Within 1 Year Before Covid-19 Vaccination

48. How many hours is the average duration of your headache if you are NOT taking attack medication for headache? *

____

Headache Within 1 Year Before Covid-19 Vaccination

49. How many hours is the average duration of your headache if you are NOT taking attack medication for headache? *

____

Headache Within 1 Year Before Covid-19 Vaccination

50. What is the average number of headache attacks per MONTH? *

- Less than one attack per month
- One attack per month or more

Headache Within 1 Year Before Covid-19 Vaccination

51. What is the average number of headache attacks per MONTH? *

____

Headache Within 1 Year Before Covid-19 Vaccination

52. Do you have a headache diagnosis made by a physician (you can choose more than one)? *

- No diagnosis
- Migraine
- Tension type headache
- Sinusitis
- Other disease

53. What is the location of your headache (you can choose more than one)? *

- [ ] On one side (always on the same side or can change sides)
- [ ] On both sides, more dominant on one side
- [ ] On both sides with same intensity

54. How was the character of the headache before the vaccination (you can choose more than one)? *

- Throbbing
- Pressing
- Jolting
- None

55. Does your head movements increase your headache (e.g. climbing stairs, walking, leaning forward)? *

- Yes
- No

56. How was the severity of your headache before the vaccination? *

- Mild, non-irritating and does not interfere with my daily work
- Moderate, uncomfortable but I can do my daily work
- Severe, I can't do my daily work

57. Which may accompany your previous headache before vaccination? (you can choose more than one) *

- [ ] Nausea
- [ ] Vomiting
- [ ] Light disturbance
- [ ] Sound disturbance
- [ ] Smell disturbance
- [ ] None

58. Do you use any regular medication other than painkillers for your headaches? *

- ( ) Yes
- ( ) No

Treatment for Headache Within 1 Year Prior to Vaccination

59. What type of medication do you use for headache treatment (you can choose more than one)? *

- [ ] Anti-depressive medication (such as Laroxyl, Efexor, Cymbalta, Cipralex)
- [ ] Anti-epileptic medicine (such as Topamax, Depakin, Convulex)
- [ ] Heart medicine (such as Dideral, Beloc, Tensinor) Sibelium
- [ ] Botox (Migraine Botox)
- [ ] Other

Treatment for Headache Within 1 Year Prior to Vaccination

60. How many DAYS you took painkillers for your headache in the last month? *

____

61. During your headaches before the vaccination would you have a different unpleasant sensation, pain or tenderness when you touch your head in a way that normally should not cause pain, when you wear glasses, when you collect your hair, when you touch somewhere etc? *

- ( ) Yes
- ( ) No

Thank you for participating in the study. Please click the Submit button to finish.
